# The loss of left atrial contractile function predicts a worse outcome in HFrEF patients

**DOI:** 10.3389/fcvm.2022.1079632

**Published:** 2023-01-11

**Authors:** Giulia Elena Mandoli, Maria Concetta Pastore, Giovanni Benfari, Martina Setti, Luca Maritan, Enrico Emilio Diviggiano, Flavio D’Ascenzi, Marta Focardi, Luna Cavigli, Serafina Valente, Matteo Cameli

**Affiliations:** ^1^Division of Cardiology, Department of Medical Biotechnologies, University of Siena, Siena, Italy; ^2^Section of Cardiology, Department of Medicine, University of Verona, Verona, Italy

**Keywords:** speckle tracking, strain, left atrium, heart failure, HFrEF, prognosis

## Abstract

**Background:**

In chronic heart failure, high intracardiac pressures induce a progressive remodeling of small pulmonary arteries up to pulmonary hypertension. At the end of left atrial conduit function, pulmonary and left heart end-systolic pressures equalization might affect left atrial systole. In this single-center prospective study, we aimed to investigate whether peak atrial contraction strain (PACS), measured by speckle tracking echocardiography, was independently associated with prognosis in heart failure with reduced ejection fraction (HFrEF).

**Materials and methods:**

Outpatients with HFrEF and sinus rhythm referred to our echo-labs were enrolled. After clinical and echocardiographic evaluation, off-line speckle tracking echocardiography analysis was performed. Primary and secondary endpoint were cardiovascular death and heart failure hospitalization, respectively. Spline knotted survival model identified the optimal prognostic cut-off for PACS.

**Results:**

The 152 patients were stratified based on PACS <8% (*n* = 76) or PACS ≥8% (*n* = 76). Patients with PACS <8% had lower left ventricle and left atrial reservoir strain and higher New York Heart Association (NYHA) class and left atrial volume index (LAVI). Over a mean follow-up of 3.4 ± 2 years, 117 events (51 cardiovascular death, 66 heart failure hospitalizations) were collected. By univariate and multivariate Cox analysis, PACS emerged as a strong and independent predictor of cardiovascular death and heart failure hospitalization, after adjusting for age, sex, left ventricle strain, and E/e’, LAVI (HR 0.6 per 5 unit-decrease in PACS). Kaplan–Meier curves showed a sustained divergence in event-free survival rates for the two groups.

**Conclusion:**

The reduction of PACS significantly and independently affects cardiovascular outcome in HFrEF. Therefore, its assessment, although limited to patients with sinus rhythm, could offer additive prognostic information for HFrEF patients.

## 1. Introduction

**FIGURE 1:**
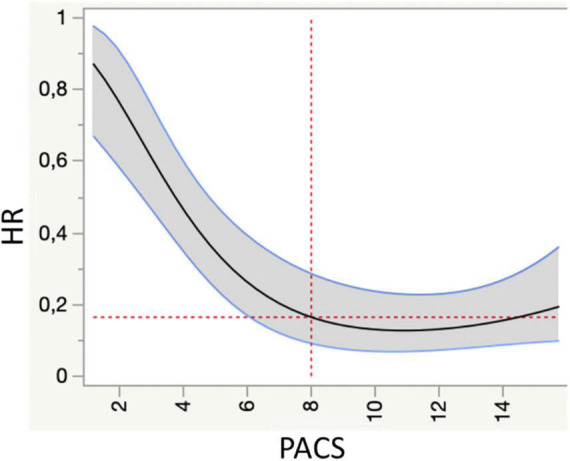
Spline knotted survival curve to assess the value of peak atrial contraction strain (PACS) as a predictor of cardiovascular mortality (primary endpoint). HR, hazard ratio.

Chronic heart failure (HF) is a progressive cardiovascular disease with increasing incidence in the last years, parallel to an increasing medium age of the population worldwide. Despite therapeutic novelties, it is still characterized by a poor prognosis, particularly in the advanced stages of the disease (1), and a high burden of symptoms, which strongly affects patients’ quality of life. Even though the classification of HF according to the European Society of Cardiology (ESC) guidelines is still based on left ventricular (LV) ejection fraction (EF), this parameter has showed a limited prognostic power, especially within the heart failure with reduced ejection fraction (HFrEF) group. Therefore, in the last years, many scientific investigations focused on the research of new echocardiographic potential prognostic markers in HFrEF, particularly after the introduction of speckle tracking echocardiography (STE) technique in daily clinical practice (2, 3). In fact, the measurement of LV strain by STE provided new insight into early diagnosis and prognostication of HF (4–6); however, in patients with chronic HFrEF including variable etiologies (such as ischemic, dilated, and hypertrophic cardiomyopathy) LV strain is often reduced being the LV the first affected chamber in these patients, so this index often lacks accuracy for risk stratification. On the other hand, left atrial (LA) reservoir strain has recently been introduced in the European association of cardiovascular imaging (EACVI) recommendations as a standard parameter in the diagnostic algorithm of diastolic function in HF with preserved ejection fraction (HFpEF) (7), due to the high amount of evidence regarding its value as early and sensitive marker of elevated LV filling pressures (8, 9) and myocardial fibrosis (10). LA strain has also showed to be a good prognostic marker both in HFrEF (11–14) and HFpEF (15, 16). However, LA reservoir strain is thought to be strictly related to LV strain in chronic HF, because of LV dysfunction in chronic increase of LV filling pressures where LV global longitudinal strain (GLS) is one of the main determinants of reservoir peak atrial longitudinal strain (PALS) (9). Importantly, LA strain could be used to measure all LA function during the cardiac cycle, reservoir one. LA contraction phase is described by peak atrial contraction strain (PACS) (Figure 1) (17). Thus, PACS may represent a more independent parameter to merely analyze LA function, being related to intrinsic atrial remodeling and residual contractile function. LA booster function could be affected in chronic HF by the establishment of pulmonary hypertension in adjunction to chronically high filling pressures, leading to pulmonary and left heart pressures equalization at the end of LA conduit phase. PACS has already shown to be a good prognostic parameter in HFrEF if measured together with PALS, however, no study has been developed on the independent value of PACS yet. The aim of our study was to assess the potential value of PACS as an independent prognostic parameter in a cohort of patients with chronic HFrEF.

## 2. Materials and methods

In this prospective single-center observational study, consecutive patients with HFrEF and sinus rhythm according to the 2016 ESC HF guidelines definition [i.e., patients with signs (pulmonary crackles, peripheral edema, elevated jugular venous pressure) and/or symptoms (dyspnea, fatigue, and ankle swelling) of HF and LV EF <40%] referred to our HF ambulatories for a cardiologic visit including echocardiography between 2015 and 2017 were enrolled. Exclusion criteria were non-sinus rhythm, previous cardiac surgery or poor acoustic window.

The patients were prospectively followed for a primary and a secondary endpoint, consisting in the occurrence of cardiovascular death and hospitalization for HF, respectively. Follow-up data were collected *via* phone calls and electronic medical records. All subjects gave their written informed consent for participation in this study. All work followed the 1975 Declaration of Helsinki. The study protocol was approved before the enrollment of the first patient by the local Ethics Committee (approval number 11757_2017).

### 2.1. Basic echocardiography

Echocardiographic examination was performed according to the EACVI/American Society of Echocardiography (ASE) recommendations for chamber quantification (18), using a high-quality ultrasound machine (Vivid E9; GE Medical System, Horten, Norway) with patients in the left lateral recumbent position.

Left ventricular and left atrial dimensions were calculated using standard views. LV ejection fraction (LVEF) and LA volume and area were assessed using the biplane modified Simpson method from the apical 4- and 2-chamber views. LV dimensions and LA volume were indexed to body surface area obtaining LV mass index and LA volume index (LAVI). From the 4-chamber view, tricuspid annular plane systolic excursion (TAPSE) was measured by M-mode; maximum early diastolic (E) and late diastolic (A) velocities were assessed by trans-mitral pulsed wave doppler to calculate E/A ratio; then, peak systolic (S’), early diastolic (E’), and late diastolic (A’) annular velocities were obtained by tissue doppler imaging, E/E’ ratio was calculated and used as index of the LV filling pressure. Mitral and tricuspid regurgitation (MR, TR) were quantified by bidimensional (2D)-echocardiography according to EACVI/ASE recommendations (18). Systolic pulmonary artery pressure (sPAP) was estimated as the sum of systolic trans-tricuspid pressure gradient and of right atrial pressure derived from the diameter and collapsibility of the inferior vena cava.

### 2.2. Speckle tracking echocardiography

Speckle tracking echocardiography analysis was performed on apical 2-, 3-, and 4- chamber images, obtained by 2D gray-scale echocardiography, with a stable electrocardiographic recording. Care was taken to obtain a good visualization of all chambers and a reliable delineation of the endocardial border. Measurements from three consecutive heart cycles were recorded and averaged. The frame rate was 60–80 frames/sec. Analysis was performed off-line by a single experienced and independent echocardiographer, who was not directly involved in the image acquisition and blinded to basic echocardiographic parameters, using a semiautomated 2D-strain software (EchoPac, GE, Milwaukee, WI, USA). The endocardial border was manually traced in apical views, delineating a region of interest (ROI) of six segments for each view. Then, necessary manual adjustments of the ROI were performed and the longitudinal strain curves for each segment were generated by the software. LV GLS was calculated as the average of 4-, 2-, and 3-chambers longitudinal strain curves. Both apical views were optimized in terms of orientation, depth, and gain to avoid LA foreshortening and to visualize the entire LA throughout the cardiac cycle. Global PALS and PACS were calculated at the end of the reservoir and the contraction phase, respectively, as the average of all LA segments in 4- and 2-chamber views, using QRS as starting point (19). In patients in whom some segments were excluded for impossible adequate tracking, strain was calculated by averaging values measured in the remaining segments.

### 2.3. Statistical analysis

Data are expressed as means ± SD (continuous variables) or as counts and percentages (binary variables).

Spline-knotted survival model was used to obtain optimal cutoff values of global PACS for the prediction of the primary endpoint (cardiovascular mortality). Using this cutoff, patients were divided into 2 groups based on the presence of PACS lower/higher than the cutoff. Differences between the groups were analyzed using Student *T*-tests for continuous variables and Chi-squared analyses for categorical variables.

Kaplan–Meier curves and Log-Rank test were used to assess the correlation of the two groups with events-free survival. Univariate and multivariate analysis were performed applying the Cox proportional hazard model to investigate the performance of global PACS as a predictor of primary and secondary endpoint; adjustment models were built using age, sex, LV strain, and E/e’, LAVI. The covariates were chosen based on their univariable association with the dependent variable as well as based on biological plausibility.

Analyses were performed using the Statistical Package for Social Sciences software, release 20.0 (SPSS, Chicago, IL, USA). *P*-values <0.05 were considered statistically significant.

## 3. Results

From an initial number of 168 patients, 152 patients were finally enrolled in this study. We excluded 8 patients for previous heart valve surgery, six because of atrial fibrillation during echocardiographic examination and two for poor acoustic window. Intra-operator reproducibility for LA strain analysis was already tested in our center. (19, 20) Mean age was 62 ± 12 years, 21% (*n* = 32) were female, mean LV EF was 30 ± 9%. All patients were receiving optimized HF therapy (according to current guidelines at the time of enrollment) including ACE inhibitors/angiotensin receptor blockers (91%), mineralcorticoid receptor antagonist (75%), betablockers (78%). Moreover, 81% of the patients had implantable cardiac device (ICD) or cardiac resynchronization therapy-defibrillator (CRT-D). Mean LA maximum volume indexed was 55 ± 18 ml/mq while LA minimum volume was 34 ± 20 ml.

Speckle tracking echocardiography analysis revealed severely reduced longitudinal deformation of both left ventricle and atrium (−8.7 ± 3.4 and 15.0 ± 6%, respectively). PACS value showed a moderate, statistically significant correlation with LA minimum volume (*r* = 0.54, *p* < 0.001).

Over a mean follow up of 3.4 ± 2 years, 117 events (51 CV death, 66 HF hospitalizations) were registered. Spline-knotted curves showed a good association of global PACS <8% with risk of cardiovascular mortality (Figure 1).

Therefore, this cut-off was used to stratify the population into two risk groups: group 1 with PACS <8% and group 2 with PACS >8%. Table 1 shows the general and echocardiographic characteristics of the study population. Patients with global PACS <8% showed lower LV GLS and global PALS, higher New York heart association (NYHA) class, N-terminal-pro-brain natriuretic peptide (NTproBNP), LAVI, and sPAP. Then, univariate and multivariate Cox analysis (see Tables 2, 3) was applied after adjusting for age, sex, LV strain, E/e’, LAVI (see Tables 2, 3 for univariate and multivariate analysis for CV death and HF hospitalization, respectively) showing a strong and independent association of global PACS reduction with the primary endpoint (HR 0.6 per 5 unit decrease in PACS).

**TABLE 1:** Clinical and echocardiographic characteristics of heart failure with reduced ejection fraction (HFrEF) patients according to PACS values.

Variable	Overall (*n* = 152)	PACS <8% (*n* = 76)	PACS ≥8% (*n* = 76)	*P*-value
Age	62 ± 12	61 ± 12	62 ± 13	0.8
Male (%, *n*)	79 (120)	82 (62)	76 (58)	0.7
BMI	27 ± 5	27 ± 5	27 ± 5	0.7
sBP (mmHg)	123 ± 21	119 ± 22	127 ± 19	0.02
HR (bpm)	70 ± 10	71 ± 11	70 ± 10	0.7
Hypertension (%, *n*)	39 (60)	34 (26)	45 (34)	0.2
Diabetes mellitus (%,*n*)	16 (25)	16 (12)	17 (13)	0.9
Dyslipidemia (%,*n*)	28 (42)	16 (12)	39 (30)	0.002
NYHA class >2 (%, *n*)	37 (56)	51 (39)	22 (17)	<0.0001
NTpro BNP (pg/L)	1,814 ± 2,059	2,294 ± 1,676	1,335 ± 2,442	0.09
LVEDVi (ml/mq)	82 ± 49	85 ± 55	80 ± 43	0.5
LVESVi (ml/mq)	58 ± 39	62 ± 43	54 ± 33	0.2
LVEF (%)	30 ± 7	28 ± 7	33 ± 7	0.0007
LAVI (ml/mq)	55 ± 18	64 ± 20	45 ± 16	<0.0001
E/A	1.6 ± 1.1	1.9 ± 1.2	1.2 ± 0.9	<0.0001
E/E’ ratio	14 ± 8	16 ± 9	12 ± 7	0.003
TAPSE (mm)	17 ± 4	17 ± 4	18 ± 5	0.2
RVFAC (%)	38 ± 9	37 ± 9	40 ± 9	0.09
sPAP (mmHg)	35 ± 11	40 ± 13	30 ± 9	<0.0001
LVGLS (%)	−8.7 ± 3.4	−7.3 ± 3.5	−10.2 ± 3.2	<0.0001
Global PALS (%)	15.0 ± 6.0	9.8 ± 4.9	20.3 ± 7.0	<0.0001

E, peak early diastolic “E” wave; E’, medium velocity of early mitral annulus descent; GLS, global longitudinal strain; LAVI, left atrial volume index; LVEDVi, left ventricular end-diastolic volume index; LVESVi, left ventricular end-systolic volume index; LVEF, left ventricular ejection fraction; PACS, peak atrial contraction strain; PALS, peak atrial longitudinal strain; RVFAC, right ventricular fractional area change, sPAP, systolic pulmonary artery pressure; and TAPSE, tricuspid annular plane excursion.

**TABLE 2:** Univariate and multivariate analysis for the prediction of cardiovascular death.

Parameter	Univariate analysis (HR)	*p*	Multivariate analysis (HR)	*p*
LAVI	1.01	0.06		
E/e’	1.03	0.07		
GLS	0.82	0.04	0.88	0.05
Age	1.00	0.49		
Male	1.14	0.52		
PACS	0.71	0.04	0.59	0.03

LAVI, left atrial volume index; GLS, global longitudinal strain; and PACS, peak atrial contraction strain.

**TABLE 3:** Univariate and multivariate analysis for the prediction of HF hospitalization.

Parameter	Univariate analysis (HR)	*p*	Multivariate analysis (HR)	*p*
LAVI	1.00	0.54		
E/e’	1.01	0.54		
GLS	0.81	0.03	1.07	0.17
Age	1.00	0.81		
Male	0.93	0.86		
PACS	0.52	<0.001	0.60	0.01

LAVI, left atrial volume index; GLS, global longitudinal strain; and PACS, peak atrial contraction strain.

**FIGURE 2:**
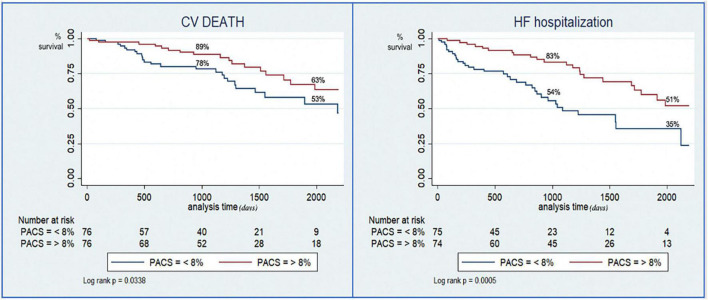
Kaplan–Meier curves for the risk stratification of cardiovascular death **(left)** and hospitalization for heart failure **(right)** based on preserved or reduced peak atrial contraction strain (PACS).

Kaplan–Meier curves showed a sustained divergence in event-free survival rates for the two groups for both the primary and the secondary endpoint (Figure 2).

## 4. Discussion

The present study was the first to show an independent prognostic value of PACS in chronic HF, identifying <8% as the optimal cut-off value for risk stratification in HFrEF.

Even though there currently is a lack of studies focused on this parameter alone, there are a lot of evidence on its association with outcome in HF and cardiomyopathies, if measured in adjunction to PALS (21–23). Moreover, Inoue et al. (9) proved that both PALS and PACS are markers of LV filling pressures in patients with reduced LV systolic function, and that PACS predicts LV filling pressures also in patients with normal LV systolic function. Lindqvist and Henein (23) showed that LA contraction strain rate was the strongest predictor of PCWP in symptomatic patients, compared to LA reservoir strain and GLS, particularly in patients with post-capillary PH and with dilated LA cavity. In our population, PACS was moderately correlated with LA minimum volume, which is probably dependent on the fact that the amount of blood remaining in the last atrium at the end of diastole is also determined by LA conduit function and LV compliance.

The rationale in considering PACS as an independent prognostic parameter in HFrEF could be deduced by the pathophysiology of chronic HF: first of all, the direct or indirect myocardial damage which causes overt LV systolic dysfunction in HFrEF and the chronic LV overload impairs myocardial contractility with severe LV GLS reduction accompanying LV EF reduction in the majority of cases. Therefore, although being important for the categorization of overall HF, none of these two parameters seems to be sensitive for risk stratification among patients with chronic systolic dysfunction. Then, the chronic increase of LV filling pressures reflects on the LA myocardium, which initially compensates for the high intracardiac pressures and eventually underwent LA wall ultrastructural changes, with remodeling and fibrosis, leading to LA dilatation and dysfunction. In these phases, LA evaluation is fundamental to assess the stage of the disease and whether the myocardial damage could be reversible or not. Therefore, PALS would be certainly reduced in a grade parallel to the age of the disease, and its value, reflecting the reservoir function in systole, would be probably influenced by LV contractile properties and longitudinal shortening. However, in the advanced phase, when the LA is not capable to face the elevated filling pressures, the transmission of hemodynamic overload on the pulmonary circulation takes place, with a chronic establishment of pulmonary hypertension, up to a point in which pulmonary and LA pressures equalized in diastole, thus affecting LA contraction and its contribution to LV filling. This represents the immediately preceding step before the transition to biventricular failure, due to severe and/or chronic pulmonary hypertension, often irreversible and requiring advance therapies.

Hence, in patients with long-standing chronic HFrEF, the analysis of LA contractile function may offer additional information for risk stratification, identifying those patients who have completely lost the LA contribution to maintain LV filling, in all the cardiac cycle phases, and who are at higher risk of transition to advanced HF.

In the light of the new therapies for HFrEF recommended in the latest ESC HF guidelines (1), there is a timely need of new indices to stratify prognosis to guide clinical choices for the management of these patients.

Considering the high feasibility and availability and low time-consumption of this parameter, the measurement of PACS could help clinicians to provide patients with HF and sinus rhythm a more tailored therapy and to decide whether to be more aggressive with medical therapy and to prescribe stricter follow up to HFrEF patients.

## 5. Limitations

Although this study shows global PACS promising results for the clinical risk stratification of HFrEF, some limitations should be encountered: first, it was a single center study conducted in a small cohort of patients with HFrEF, therefore, large-scale studies should confirm our findings.

Moreover, PACS was investigated as an independent parameter and was not compared with PALS, which may be associated with it in some way. Finally, an intrinsic limitation of the parameter led to the exclusion of patients with atrial fibrillation, which is a common condition in HF, since PACS is not feasible in these patients.

## 6. Conclusion

In conclusion, in patients with chronic HFrEF, LA contraction might be affected because of the chronic hemodynamics overload. The assessment of impaired LA contraction as a reduction of global PACS, acquired by speckle tracking echocardiography, significantly and independently affects CV outcome in HFrEF. Therefore, although limited to patients with sinus rhythm, the evaluation of global PACS could provide additive information for risk stratification of HFrEF patients.

## Data availability statement

The raw data supporting the conclusions of this article will be made available by the authors, without undue reservation.

## Ethics statement

The studies involving human participants were reviewed and approved by the Comitato Etico Regionale per la Sperimentazione Clinica della Regione Toscana Sezione: AREA VASTA SUD EST. The patients/participants provided their written informed consent to participate in this study.

## Author contributions

GM: conceptualization, methodology, software, formal analysis, writing—original draft, and supervision. MP: software, formal analysis, and investigation. GB: data curation and investigation. MS, LM, LC, and SV: data curation. ED: writing—review and editing, validation, and visualization. MF: investigation. FD’A: conceptualization and methodology. MC: conceptualization, methodology, software, and project administration. All authors have participated in the work and have reviewed and agreed with the content of the article.
